# Validated HPLC method for quantification of copanlisib in mice plasma: application to a pharmacokinetic study

**DOI:** 10.5599/admet.782

**Published:** 2020-03-04

**Authors:** Ashok Zakkula, Pavan Kumar Kurakula, Sreekanth Dittakavi, Prasanthi Daram, Ram Murthi Bestha, Mohd Zainuddin, Ravi Kumar Trivedi, Ramesh Mullangi

**Affiliations:** 1 Drug Metabolism and Pharmacokinetics, Jubilant Biosys Ltd, Industrial Suburb, Yeshwanthpur, Bangalore-560 022, India; 2 Department of Pharmacology, Raghavendra Institute of Pharmaceutical Education and Research, Anantapur-515721, A.P, India

**Keywords:** Copanlisib, HPLC, method validation, mice plasma, pharmacokinetics

## Abstract

Copanlisib is a pan phosphatidylinositol 3-kinase (PI3K) inhibitor approved for follicular lymphoma. In this paper, we present the data of development and validation of a high-performance liquid chromatography (HPLC) method for the quantitation of copanlisib in mice plasma as per the FDA regulatory guideline. The method involves the extraction of copanlisib along with internal standard (IS, enasidenib) from mice plasma (100 μL) using ethyl acetate as an extraction solvent. The chromatographic resolution of copanlisib and the IS was achieved on a Hypersil Gold C_18_ column maintained at 40 °C using a binary gradient mobile phase [10 mM ammonium formate (pH 4.0) and acetonitrile]. The flow-rate was 0.8 mL/min. For the detection of copanlisib and the IS, the photo-diode array detector was set at λ_max_ 310 nm. Copanlisib and the IS eluted at 6.60 and 7.80 min, respectively with a total run time of 10 min. The calibration curve was linear over a concentration range of 50 to 5000 ng/mL for copanlisib (r^2^≥ 0.998). The results of intra- and inter-day accuracy and precision studies were within the acceptable limits. Copanlisib was stable on bench-top, in auto-sampler, up to three freeze/thaw cycle and long-term storage at -80 °C. The application of the validated method was shown in a mice pharmacokinetic study.

## Introduction

**Figure 1.**
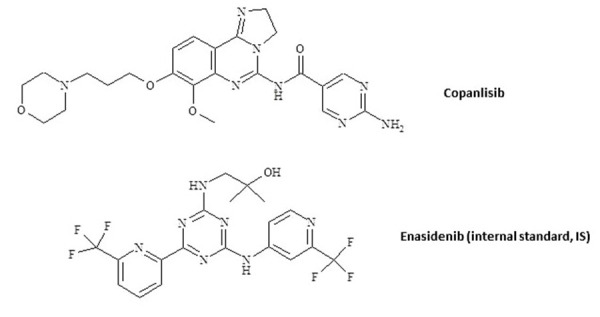
Structural representation of copanlisib and enasidenib (internal standard, IS)

Phosphoinositide 3-kinases (PI3Ks) are lipid kinases, which play a pivotal role in cell cycle, cellular metabolism, apoptosis etc. along with α-serine/threonine-protein kinase (AKT)/mammalian target of rapamycin (mTOR) pathway [1]. Abnormal activation of PI3K pathway has been shown to drive tumorigenesis [2]. Among three classes of the PI3K enzyme, class I PI3K is linked with malignance and it exists in four isoforms: α, β, γ and δ [3, 4]. PI3K inhibitors represent a novel class of targeted therapies for the treatment of human malignancies [5]. Preclinical and clinical studies demonstrated that inhibition of PI3K is an effective therapeutic strategy for lymphomas treatment [6]. Copanlisib (Fig. 1; BAY 80-6946), is a pan-PI3K inhibitor with predominant activity against PI3K-α and PI3K-δ forms, which are expressed in malignant B-cells. The IC_50_ (half-maximal inhibitory concentration) against PI3K α, β, γ and δ isoforms was 0.5, 3.7, 6.4 and 0.7 nmol/L, respectively [7]. It induces tumor cell death by apoptosis and inhibition of proliferation of primary malignant B cell lines. Copanlisib was approved for treatment of follicular lymphoma as an *intravenous* infusion for adults. In clinic, at 0.8 mg/kg (*intravenous* infusion), copanlisib showed promising efficacy in patients with solid tumors and hematological malignancies. Across the dose range of 0.1-1.2 mg/kg, it has shown dose proportional increase in *C*_max_ (maximum concentration in plasma) and AUC_0-t_ (area under curve from time zero to last measurable time point). Typically, *C*_max_ attained between 0.5-1.0 h (*T*_max_). The human plasma protein binding of copanlisib is ~84%. Copanlisib was majorly metabolized by CYP3A4 (>90% metabolism) and to some extent by CYP1A1 (~10% of metabolism) to yield M1, an active metabolite, which possess similar potency as copanlisib. The terminal half-life and clearance for copanlisib at 0.8 mg/kg were 39.1 h and 18.9 L/h. Copanlisib has high volume of distribution (871 L). Up to 50% of unchanged drug and remaining amount in the form of metabolites is excreted in humans [8].

Until date, only one bioanalytical method was published for quantification of copanlisib [9]. In the reported LC-MS/MS (liquid chromatography coupled with tandem mass spectrometry) method, authors used one step liquid-liquid extraction method for mice plasma samples processing. The linearity range was 3.59-3588 ng/mL. Using an isocratic mobile phase, copanlisib and the internal standard were resolved on a HyPurity C_18_ column having a total run time of 3.0 min.

Although LC-MS/MS is a powerful tool for the quantitation of drugs in various biological matrices with higher sensitivity in short run time, its high cost and maintenance limit its availability for most of the hospitals, academic institutes and research laboratories. The lower cost and affordability of the HPLC instrument compared to that of LC-MS/MS renders HPLC methods more eligible for wide routine use.

In clinic, at efficacy dose of 0.8 mg/kg, copanlisib plasma concentrations were ~50 ng/mL at 8 h (both on day-1 and day-15) in patients with non-Hodgkin’s lymphoma and advanced solid tumors [10, 11]. By achieving 50 ng/mL sensitivity for copanlisib on HPLC-UV, we believe our present method can be used in hospitals for routine therapeutic drug monitoring of copanlisib. Besides, the proposed method can also be in research laboratories for routine pharmacokinetic and/or toxicokinetic studies samples analysis. In order to ensure the reliability, reproducibility and sensitivity of the method, the developed analytical method was validated for various parameters in accordance with FDA guideline. The validated method was applied to investigate the pharmacokinetics of copanlisib post *intravenous* administration to mice.

## Materials and methods

### Chemicals and reagents

Copanlisib (purity: 99.7%) was obtained from Beijing Yibai Biotechnology Co., Ltd, Beijing, China. Enasidenib (purity: 98%) used as an internal standard (IS) was purchased from Aaron, Shanghai, China. Solutol, ethanol and dimethyl sulfoxide (DMSO) were purchased from Sigma-Aldrich, St. Louis, MO, USA. HPLC grade acetonitrile and methanol were purchased from J.T. Baker Avantor, PA, USA. Analytical grade hydrochloric acid and ammonium formate were purchased from S.D. Fine Chemicals, Mumbai, India. All other chemicals and reagents were of analytical grade and used without further purification. The control mice K2.EDTA plasma was procured from Animal House, Jubilant Biosys, Bangalore.

### HPLC operating conditions

Analysis of copanlisib in plasma samples was performed on a Waters 2695 Alliance HPLC system (Waters, Milford, USA) equipped with performance PLUS inline degasser along with an auto-sampler, column oven and photo diode array (PDA) detector set at *λ*_max_ 310 nm. Chromatographic resolution of copanlisib and the internal standard (IS) was achieved by injecting 25 μL of the processed sample on a Hypersil Gold C_18_ column (250 × 4.0 mm, 5 μm; Thermo Scientific, USA) maintained at 40±1 °C using a binary gradient mobile phase consisting 10 mM ammonium formate, pH: 4.0 (adjusted with formic acid) (solvent A) and acetonitrile (solvent B) delivered at a flow-rate of 0.8 mL/min. Initial eluent composition was 90% A, maintained for 5.0 min, and followed by a linear 0.5 min ramp to 10% A, which was maintained for until 5.5 min. The mobile phase composition returned to 90% A at 8.0 min. Equilibration time was 2.0 min.

### Preparation of stock solutions for copanlisib and the IS

For the preparation of calibration curve (CC) and quality control (QC) samples, two primary stock solutions of copanlisib were made at 1.0 mg/mL in 0.1 N HCl:DMSO (2:98, v/v). Due to copanlisib limited solubility in organic solvents we have used 0.1 N HCl. The primary stock solution of the IS (1.0 mg/mL) was prepared in DMSO and subsequently diluted with 80% methanol to get work stock at 0.60 μg/mL. The primary stock solutions of copanlisib and the IS were stored at -20±5 °C, which were found to be stable for 50 days.

### Preparation of calibration curve standards and quality control samples

The first set of primary stock solution of copanlisib was diluted appropriately and subsequently used to prepare a calibration curve (CC) standards. The calibration standard samples were made by spiking the blank mice plasma (90 μL) with each corresponding working solution of copanlisib (10 μL) thereby yielding final concentrations of 50, 100, 500, 750, 1250, 2500, 3750 and 5000 ng/mL.

For the determination of precision and accuracy, samples were prepared by spiking blank mice plasma in bulk with the second working stock solution of copanlisib at appropriate concentrations and 100 μL aliquots were distributed into different tubes. The QCs prepared were: 50 ng/mL (lower limit of quantification quality control; LLOQ QC), 150 ng/mL (low quality control; LQC), 2250 ng/mL (medium quality control; MQC) and 3500 ng/mL (high quality control; HQC). All the QCs were stored together at  -80±10 °C until analysis.

### Sample preparation

To an aliquot of 100 μL mice plasma sample, 1.0 mL of ethyl acetate was added and vortex mixed for 3 min; followed by centrifugation for 5 min at 14,000 rpm in a refrigerated centrifuge (Eppendorf 5424R) maintained at 5 °C. The organic layer (800 μL) was separated and evaporated to dryness at 50 °C using a gentle stream of nitrogen (Turbovap®, Zymark®, Kopkinton, MA, USA). The residue was reconstituted in 100 μL of the IS solution (600 ng/mL) and 25 μL was injected onto HPLC system for analysis.

### Validation procedures

A full validation according to the US FDA guidance was performed for the quantitation of copanlisib in mice plasma [12].

The selectivity of the proposed method was assessed by evaluating the presence of interfering the peaks at the retention times of copanlisib and the IS in six different batches of blank mice plasma samples. The auto-injector carry over was determined by injecting the highest calibration standard (5.0 μg/mL) followed by injection of mice plasma blank samples. Recovery of copanlisib was determined by comparing their respective response from QCs (LQC and HQC) after the extraction process against their non-extracted samples. Recovery of the IS was determined at 600 ng/mL. Intra- and inter-day accuracy and precision were determined at four QC levels [LLOQ QC (50 ng/mL), LQC (150 ng/mL), MQC (2250 ng/mL) and HQC (3500 ng/mL)] along with calibration curve (0.05-5.00 μg/mL). Stability (auto-sampler, bench-top, freeze-thaw and long-term) studies, dilution effect and incurred sample reanalysis (ISR) were also evaluated as per regulatory guideline requirement [12].

### Pharmacokinetic study in mice

Twelve male Balb/C mice (weight range: 26-28 g) were procured from Vivo Biotech, Hyderabad, India. Animal study protocol used in this study was approved by the Institutional Animal Ethics Committee, Jubilant Biosys (IAEC/JDC/2019/188R). Mice were housed for a period of seven days having free access to feed and water before the pharmacokinetic study. Mice received copanlisib *intravenously* [2% 0.1 N HCl, 10% DMSO, 10% Solutol:absolute alcohol (1:1, v/v) and 78% normal saline; strength: 0.5 mg/mL; dose volume: 10 mL/kg] at 5.0 mg/kg as a bolus dose. Blood samples (100 μL) were collected at pre-determined time points (0.12, 0.25, 0.5, 1, 2, 4, 8 and 24 h) through retro-orbital plexus (using Micropipettes, Drummond Scientific, PA, USA; catalogue number: 1-000-0500) into polypropylene tubes (having K_2_.EDTA as an anti-coagulant). Sparse sampling technique (three mice per time point) was adopted during blood collection so that blood loss from each mouse was kept less than 10% of total blood volume. Plasma was harvested by centrifuging the blood using Biofuge (Hereaus, Germany) at 1760 g for 5 min and stored frozen at -80±10 °C until analysis. Mice were allowed to access feed 2 h post-dosing.

### Pharmacokinetic analysis

Pharmacokinetic parameters were calculated by a non-compartmental method using Phoenix WinNonlin 8.1 software (Pharsight, Mountain View, CA, USA). Key pharmacokinetic parameters like extrapolated plasma drug concentration at time zero following *intravenous* bolus injection (*C*_0_), area under the curve from time zero to infinity (AUC_0-∞_), volume of distribution (*V*_d_), total body clearance (CL) and half-life (*t*_½_) were determined for copanlisib.

## Results and discussion

### Method development and optimization

Several trials were taken with various columns, mobile phase compositions to select the chromatographic conditions, which will give a good resolution of copanlisib and the IS from the endogenous matrix substances within a suitable run time. Several mobile phases were tried by changing the combination of different organic solvents (acetonitrile and methanol) and buffers (eg: formic acid, ammonium acetate, phosphate buffer etc.) with altered flow-rates (in the range of 0.60-1.20 mL/min). To choose a stationary phase a variety of columns namely X-Terra Phenyl, Atlantis, Hypersil Gold C_18_ were evaluated. Our trials revealed that mobile phase comprising 10 mM ammonium formate (pH 4.0):acetonitrile delivered in a gradient program at a flow-rate of 0.8 mL/min on Hypersil Gold C_18_ column gave a stable base line with good resolution between copanlisib and the IS with a total run time of 10 min with no interference of endogenous plasma peaks. The UV detector was set at *λ*_max_ 310 nm. For the optimized conditions, enasidenib was found to be a suitable internal standard as it exhibited good resolution, retention time and UV absorbance intensity (UV *λ*_max_ 287 nm) at the same wave length of copanlisib.

### Method validation

**Figure 2.**
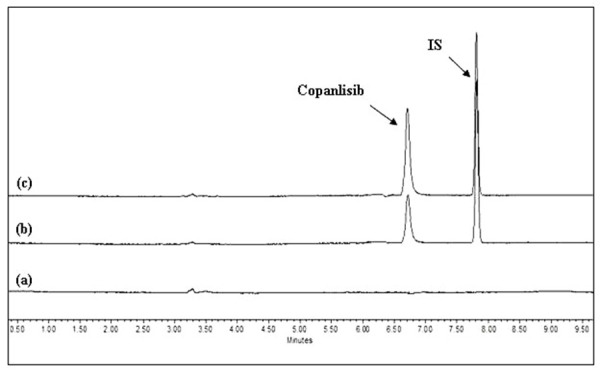
HPLC chromatograms of a 25 μL injection of (a) blank mice plasma (b) blank mice plasma spiked with copanlisib (LLOQ: 50 ng/mL) along with the IS (c) a 0.5 h plasma sample showing the peak of copanlisib (concentration: 3500 ng/mL) following *intravenous* administration of copanlisib to mice at 5.0 mg/kg.

With protein precipitation technique the recovery of copanlisib and IS was very poor (<40%). Liquid-liquid extraction with ethyl acetate gave best results in terms of extraction recovery, reproducibility and cleaner samples. The mean ± S.D recovery of copanlisib at LQC and HQC was 83.8 ± 4.51 and 83.9 ± 1.17%, respectively. The recovery of the IS was 99.5 ± 1.33%. As shown in Fig. 2, both copanlisib and the IS peaks were well resolved and no interference at the retention times of copanlisib and the IS from the endogenous components of mice. The retention time of copanlisib and the IS was 6.60 and 7.80 min, respectively. The calibration curves (n=4) for copanlisib were observed to be linear in the range of 50-5000 ng/mL. A representative equation for the calibration curves is as follows: y = 0.0005 x + 0.003. A regression equation with a weighting factor of 1/*X*^2^ of each drug to the IS concentration was found to produce the best fit for the concentration-detector response relationship. The correlation coefficients (r^2^) were more than 0.999, indicating an acceptable linearity of our method. The accuracy observed for the mean of back-calculated concentrations for four calibration curves was within 89.3-109%; while the precision (%RE) values ranged from 0.95-1.05%. We did not observe any carry-over produced by the highest calibration sample on the following injected mice blank plasma extracted sample for copanlisib.

**Figure 3.**
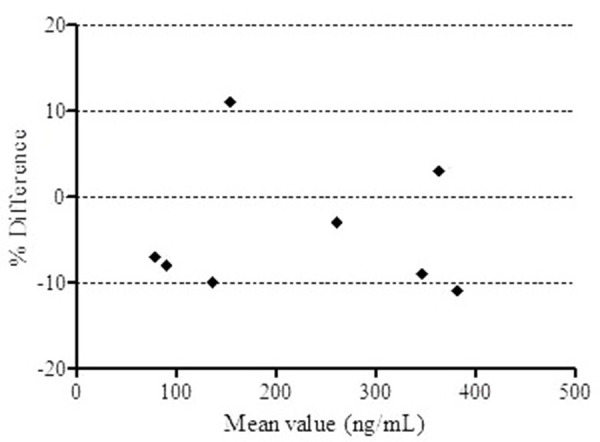
Bland-Altman plot showing the incurred sample reanalysis (ISR) data for copanlisib

Accuracy and precision data for intra- and inter-day mice plasma samples determined for copanlisib (from four different batches) at LLOQ QC (50 ng/mL), LQC (150 ng/mL), MQC (2250 ng/mL) and HQC (3500 ng/mL) are presented in Table 1. The intra- and inter-day precisions (RSD) were within 7.59%, and accuracy (RE) ranged between 0.97-1.07%. The assay values on both the occasions (intra- and inter-day) were found to be within the accepted variable limits indicating that the present method is reproducible, accurate and precise. Table 2 summarizes the results of stability studies conducted for copanlisib in mice plasma. The measured concentrations for copanlisib at LQC (150 ng/mL) and HQC (3500 ng/mL) deviated within ±15% of the nominal concentrations in a battery of stability tests namely in-injector (16 h), bench-top (6 h), repeated three freeze/thaw cycles and freezer stability at -80±10 °C for 30 days (Table 2) supported the stability of copanlisib at various stability conditions. The dilution integrity was confirmed for QC samples that exceeded the upper limit of the quantitation (ULOQ) of calibration curve (up to 35000 ng/mL). The mean accuracy and precision for 15 times diluted samples were found to be less than 7.62 and 6.25%, respectively, which show the ability to dilute samples up to a dilution factor of ten in a linear fashion (Table 2). All the samples selected for ISR met the acceptance criteria. The back-calculated accuracy values ranged between 89.2-110% from the initial assay results as shown in Fig. 3 using a Bland-Altman plot.

### Pharmacokinetic study

Plasma samples collected during pharmacokinetic study were thawed at room temperature and processed as mentioned in Sample preparation section. Along with plasma samples, LQC, MQC and HQC samples (made in blank plasma) were assayed in duplicate and were distributed among unknown samples in the analytical run. Plasma samples showed high concentration above the high calibration standard (5.00 μg/mL) were diluted appropriately with mice blank plasma to bring the concentration within linearity range. The criteria for acceptance of the analytical runs encompassed the following: (i) ≥67% of QC samples should be ±15% of the nominal concentration values (ii) ≥ 50% of QC samples per level should be ±15% of their nominal concentration values [12].

**Figure 4.**
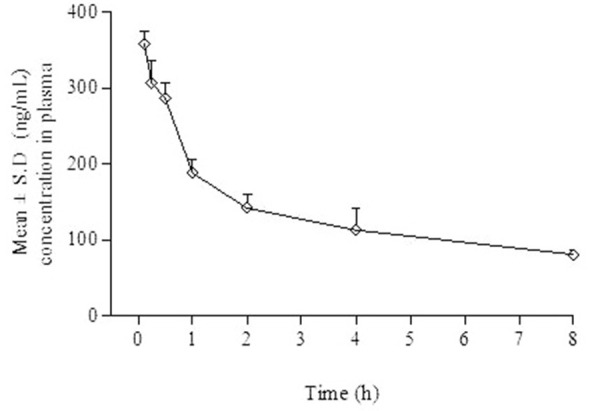
Mean plasma concentration *versus* time profile for copanlisib in mice plasma following *intravenous* administration to mice.

In the mice, plasma concentrations of copanlisib decreased mono-exponentially after *intravenous* administration. Fig. 4 depicts the mean ± S.D plasma concentrations versus time for copanlisib following *intravenous* administration to mice at 5.0 mg/kg. Copanlisib was quantifiable up to 8.0 h post *intravenous* administration to mice. Post *intravenous* administration, the CL and *V*_d_ were found to be 42.1 mL/min/Kg and 27.6 L/kg, respectively. The AUC_0-∞_ attained post *intravenous* administration was 1978 (ng h)/mL. The *t*_½_ was 7.58 h. In summary the validated method was sensitive enough to calculate the pharmacokinetic parameters of copanlisib.

Patnaik et al. [10] and Kim et al. [11] reported the plasma concentrations of copanlisib post administration at efficacy dose (0.8 mg/kg as 1 h *intravenous* infusion) to patients with advanced solid tumors and non-Hodgkin’s lymphoma. Plasma samples collected in these studies on day-1 and day-15 were analyzed using an LC-MS/MS method. We have digitalized the reported plasma concentrations versus time plots of copanlisib reported by Patnaik et al. [10] and Kim et al. [11] using DigitizeIt software [13] and found that in both studies copanlisib showed ~50 ng/mL till 8 h. By achieving 50 ng/mL sensitivity for copanlisib on HPLC-UV in the present method, we believe our present method can be reliably used in hospitals for routine therapeutic drug monitoring of copanlisib. By increasing the plasma volume for sample processing and injection volume for HPLC analysis, there is a great possibility that our validated HPLC-UV can be used to quantify the copanlisib plasma concentration at terminal time points at therapeutic doses.

## Conclusions

A simple reversed-phase HPLC method for determination of copanlisib in mice plasma has been developed and validated. The proposed method is highly specific, accurate, precise and reproducible. All the validation parameters were within the acceptable limits for a bioanalytical method as per regulatory guideline. This method has been successfully applied to a pharmacokinetic study in mice.

## Figures and Tables

**Table 1.** Intra- and inter-day precision and accuracy determination of copanlisib quality controls in mice plasma

Theoretical concentration (ng/mL)	Batch	Measured concentration (ng/mL)		
		Mean ± SD	RSD	Accuracy, %
**Intra-day** (Six replicates at each concentration)				
50.0	1	49.4 ± 4.76	9.63	98.9
	2	50.8 ± 3.89	7.65	101
	3	54.3 ± 1.35	2.49	108
	4	51.0 ± 3.26	6.53	103
150	1	139 ± 1.95	1.39	93.2
	2	151 ± 5.77	3.80	101
	3	146 ± 2.88	1.98	97.3
	4	145 ± 3.46	2.36	97.3
2250	1	2155 ± 46.5	2.16	95.8
	2	2120 ± 78.9	3.72	94.2
	3	2297 ± 38.1	1.66	102
	4	2190 ± 54.1	2.50	97.4
3500	1	3460 ± 81.2	2.32	112
	2	3507 ± 70.0	2.00	104
	3	3545 ± 53.3	1.50	105
	4	3516 ± 68.1	1.94	107
**Inter-day** (Twenty four replicates at each concentration)				
50.0		51.2 ± 3.91	7.59	103
150		146 ± 6.22	4.28	97.3
2250		2190 ± 95.1	4.35	96.9
3500		3504 ± 72.0	2.06	99.9

RSD: relative standard deviation (SD × 100/Mean)

RE: relative error (measured value/actual value)

SD: standard deviation

**Table 2.** Stability and dilution integrity data for copanlisib in mice plasma

Experiment	Spiked concentration (ng/mL)	Measured concentration (ng/mL) Mean ± SD^a^ (n = 6)	Accuracy (%)^b^	Precision (% CV)
Bench-top (6 h) stability	150	150 ± 2.72	99.8	1.80
	3500	3548 ± 66.6	101	1.88
In-injector (16 h) stability	150	146 ± 4.77	98.0	3.25
	3500	3808 ± 34.0	107	0.89
Freeze-thaw (3 cycles) stability	150	159 ± 12.3	106	7.70
	3500	3774 ± 107	107	2.84
Long-term stability at -80°C (30 days)	150	146 ± 3.44	97.3	2.35
	3500	3758 ± 23.0	106	0.61
*Dilution integrity	2333	2522 ± 403	108	6.25

RSD: relative standard deviation (SD x 100/Mean)

RE: relative error (measured value/actual value)

*Plasma samples prepared at 35,000 ng/mL (10-fold above HQC) diluted with blank plasma by 15-fold and analyzed
